# Facial Trustworthiness Judgments in Children with ASD Are Modulated by Happy and Angry Emotional Cues

**DOI:** 10.1371/journal.pone.0097644

**Published:** 2014-05-30

**Authors:** Frances Caulfield, Louise Ewing, Nichola Burton, Eleni Avard, Gillian Rhodes

**Affiliations:** Australian Research Council Centre of Excellence in Cognition and its Disorders, School of Psychology, University of Western Australia, Perth, Western Australia, Australia; The George Washington University, United States of America

## Abstract

Appearance-based trustworthiness inferences may reflect the misinterpretation of emotional expression cues. Children and adults typically perceive faces that look happy to be relatively trustworthy and those that look angry to be relatively untrustworthy. Given reports of atypical expression perception in children with Autism Spectrum Disorder (ASD), the current study aimed to determine whether the modulation of trustworthiness judgments by emotional expression cues in children with ASD is also atypical. Cognitively-able children with and without ASD, aged 6–12 years, rated the trustworthiness of faces showing happy, angry and neutral expressions. Trust judgments in children with ASD were significantly modulated by overt happy and angry expressions, like those of typically-developing children. Furthermore, subtle emotion cues in neutral faces also influenced trust ratings of the children in both groups. These findings support a powerful influence of emotion cues on perceived trustworthiness, which even extends to children with social cognitive impairments.

## Introduction

The face plays an important role in social cognition, with people routinely inferring personality traits from an individual’s appearance [1]. Evaluations of an individual’s trustworthiness are one of the many important appearance-based trait inferences made on a daily basis [2]. Although not necessarily accurate, adults make reliable, automatic trustworthiness attributions after very brief exposure to faces and these judgments can have a substantial impact on social interactions, influencing how people behave towards others [3]–[6].

There has recently been increasing interest in trait inferences in Autism Spectrum Disorder (ASD), which is characterised by social cognitive impairments [7]. Individuals with ASD often have difficulties understanding the mental state of others and reading social information from faces [8]–[10], which may be critical for these appearance-based trait inferences. Trustworthiness judgments are of particular interest in this group because imaging studies have revealed that the amygdala, which is involved in trust perception [11]–[13], may be atypical in individuals with ASD [8], [14]–[16].

Current findings regarding facial trustworthiness judgments in ASD are mixed. Some studies report no significant differences in trustworthiness judgments between typical adults and adults with ASD [17]–[19]. Other studies report that individuals with ASD significantly overrate the trustworthiness of negatively valenced faces [11], [20], [21]. Critically however, in these latter studies the responses of adults with ASD are highly variable and overlap considerably with the responses of the typical adults [11] and in one study the group differences did not survive correction for multiple comparisons [20]. Taken together, these findings provide limited support for differences in facial trust perception between adults with and without ASD. Still, there may be other atypicalities in responses to trustworthy and untrustworthy faces in individuals with ASD. For example, there have been some reports of atypical neural [18] and autonomic [17] responses in adults with ASD during trust evaluation tasks.

A critical feature of trust perception that is yet to be investigated in individuals with ASD is the role of emotional expression cues. Researchers propose that facial expressions of emotion may convey not only an individual’s current affective state, but also more stable trait impressions, such as trustworthiness, consistent with that emotion [22]–[24]. In particular, happy and angry expressions have been associated with perceived trustworthiness, with happy expressions found to increase the appearance of trustworthiness and angry expressions found to diminish it [25]–[30]. When these expressions are overtly expressed the process is known as temporal extension [24]. When these expressions are very subtle, such as those perceptible in neutral faces, the phenomenon is known as emotion overgeneralization [31]. There is also evidence that this association between perceived trustworthiness and facial cues to emotion is bidirectional. For example, computer-modelling studies have shown that manipulating the trustworthiness of faces influences their perceived emotional expressions [26]. These findings suggest a strong association between emotional expressions and perceived facial trustworthiness.

The same may not be the case however for individuals with ASD, who often demonstrate difficulties extracting emotional information from faces. Indeed the Diagnostic and Statistical Manual of Mental Disorders-IV (DSM-IV) criteria for ASD include items related to deficits in identifying and processing emotion, such as the use of facial expressions, lack of emotional sharing and impaired responses to others emotions [7]. The extent of emotion processing deficits in ASD remains a topic of much debate [32], [33]–[35], but the conclusions of a recent meta-analysis of 48 studies support a general impairment in emotion recognition in individuals with ASD [32]. Given the association between perceived emotion and trustworthiness, it seems plausible that difficulties reading emotional information from faces could disrupt the typical modulation of trust inferences by expression cues.

The current study aimed to directly examine the influence of emotional expressions on trust judgments in children with ASD. Children with and without ASD, aged 6 to 12 years, made trustworthiness ratings of neutral faces and faces displaying happy and angry expressions. We created two intensity levels of overtly expressed happiness and anger (25% and 50%) and looked for evidence of temporal extension. We also looked for evidence of overgeneralization of emotion cues present in neutral expression faces. Temporal extension and emotion overgeneralization have both been shown to occur in trust judgments of typical adults [26]–[30], older adults [25] and typically-developing children [36]. Here, we were interested to see whether these effects also extend to children with ASD. Specifically, we were interested in whether cues to anger diminish the appearance of trustworthiness for children with ASD and whether cues to happiness increase it.

We also measured expression recognition ability to assess whether any differences in the modulatory effects of emotion are related to differences in expression sensitivity between our two participant groups. Evidence of impaired expression processing *and* reduced modulation of trust judgments by expression in children with ASD could signal that expression recognition is critical for trustworthiness attribution. In contrast, evidence of intact expression processing without modulation of trust judgments could suggest that children with ASD have atypical sensitivity to the social affordances of expression cues.

## Method

### Ethics Statement

**Figure 1:**
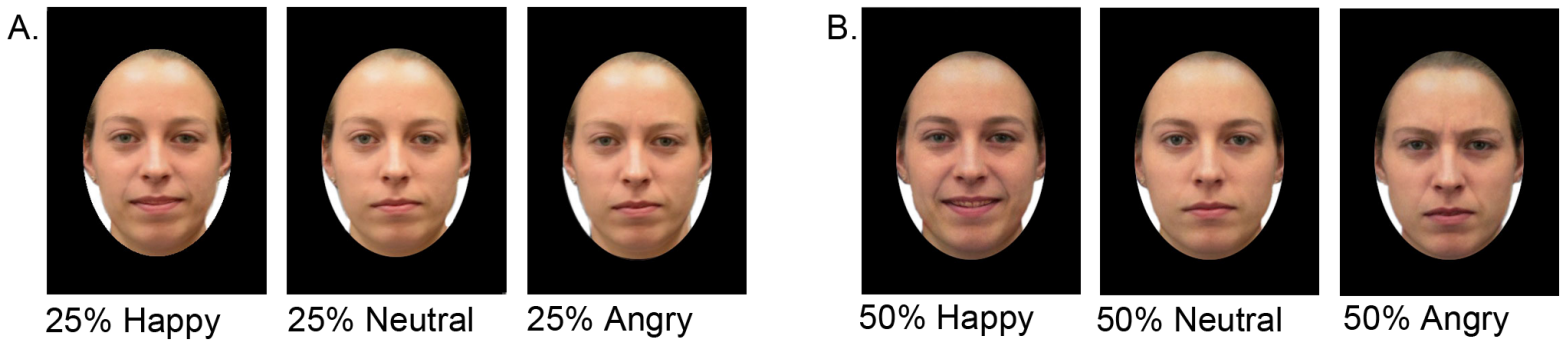
An example of expression stimuli used in the trust rating task. A happy, neutral and angry expression at 25% intensity (A) and 50% intensity (B). The identity displayed here did not appear in the stimulus set.

This study was approved by the Human Research Ethics Committee at the University of Western Australia and all parents provided written consent prior to their child’s participation in the project. All children also gave verbal and written consent before taking part. The individual displayed in figure 1 of this manuscript has given written informed consent (as outlined in PLOS consent form) to publish a recognizable image.

### Participants

Fifteen cognitively-able boys with ASD aged 6 years 9 months to 12 years 8 months (*M* = 9∶3, *SD = *1∶10) were recruited from the West Australian Register for Autism Spectrum Disorders, local schools and community groups. They had all received an independent diagnosis of an ASD by a multidisciplinary team following DSM-IV criteria [37]. Parents completed the Social Communication Questionnaire (SCQ) [38], a retrospective questionnaire measure of their children’s autism symptomatology. All children scored at or above the cut-off for clinically significant levels of autistic symptomatology (score of 12) [39]. Children also completed Module 3 of the Autism Diagnostic Observation Schedule-2 (ADOS-2) [40], with six participants scoring below the cut-off (score of 7) on this semi-structured standardized assessment of current autism symptomatology. Note - When the data were reanalysed including only those children with ASD who scored above the ADOS-2 cut-off for *current* ASD symptoms the pattern of results remained unchanged (see Appendix S1).

In addition, 15 typically-developing children (8 male) aged between 6 years 3 months and 11 years 6 months (*M* = 8∶10, *SD = *1∶9) were recruited from local schools and community groups. These children were well matched to the children with ASD on chronological age, non-verbal IQ and full-scale IQ, and did not differ significantly in terms of verbal IQ (see Table 1). Six additional children were excluded prior to matching due to inattention (ASD* = *1, Typical = 1) or difficulties understanding the concept of trustworthiness (ASD = 2, Typical = 2) during testing. No typically-developing child displayed clinically significant levels of ASD symptomatology, as indicated by scores on the SCQ. Face recognition was significantly impaired in the children with ASD relative to this typical group (see Table 1), as indexed by scores on the Cambridge Face Memory Test – for Children (CFMT-C) [41].

**Table 1:** Descriptive statistics for age, cognitive ability and autism symptomatology measures.

Measure	Group					
	ASD (n = 15)		Typical (n = 15)			
	*M (SD)*	Range	*M (SD)*	Range		Cohen’s *d*
Age (months)	110.9 (21.6)	81–152	106.0 (20.9)	75–138	*t*(28) = −.63, *p* = .54	0.24
Nonverbal IQ^a^	107.8 (17.6)	84–134	105.3 (13.4)	81–129	*t*(28) = −.43, *p* = .67	0.16
Verbal IQ^a^	96.5 (11.1)	81–118	102.1 (7.3)	87–114	*t*(24.22) = 1.64, *p* = .12^e^	0.67
Full Scale IQ^a^	101.6 (12.7)	87–125	104.0 (6.5)	88–112	*t*(20.89) = .65, *p* = .52^e^	0.28
SCQ^b^	25.5 (6.8)	12–34	1.7 (2.4)	0–8	*t*(17.41) = −12.84, *p*<.001^e^	6.16
ADOS-2^b^^,^ c	7.9 (5.3)	0–21				
CFMT-C^d^	69.3 (9.1)	52–82	77.1 (12.1)	57–97	*t*(28) = 1.99, *p* = .03^f^	0.75

a Nonverbal and verbal IQ were measured with the WASI [65]: Matrix Reasoning and Block Design (nonverbal IQ) and Similarities and Vocabulary (verbal IQ). Full-scale IQ (FSIQ) was derived by standardizing the sum of both verbal and performance ability scores against age-based norms.

b Higher scores on both the SCQ [38] and ADOS-2 [40] indicate a greater degree of autism symptomatology.

c ADOS-2 score reported = Communication+Social Interaction algorithm total (cut-off = 7).

d Accuracy (total percentage correct) on the Cambridge Face Memory Test - for Children [41].

e Equal variances not assumed.

f One-tailed independent samples t-test.

An additional 16 adults (18–54 years, *M = *29.1, *SD* = 10.9; 4 male) were recruited to rate the neutral face stimuli from the trust rating task on their resemblance to happy and angry expressions.

### Procedure

The trust rating task and expression recognition task were run on a 15-inch MacBook Pro laptop computer and were part of a larger battery of unrelated tasks conducted with participants over two or three 90–120 minute activity sessions in the family home, school or at the University of Western Australia. The experimenter sat alongside the child throughout all tasks to monitor engagement and provide verbal encouragement.

#### Trust ratings

Participants rated the trustworthiness of faces displaying happy, angry and neutral expressions. Children were told that an Alien named Zeb needed help to complete a mission to learn more about human trustworthiness. They were given a brief description of trustworthiness that focused on three key elements of trust: honesty, reliability and emotional trust [42], [43]. They then answered three questions to confirm they understood our operationalization of trustworthiness: Sarah watched her little brother like she promised. Would you trust Sarah?; Jake copied the test answers from the person sitting next to him. Would you trust Jake?; Mia hasn’t told anyone that her best friend is afraid of the dark. Would you trust Mia? If participants responded incorrectly to any item, we repeated our description of trust and repeated all three questions (one child with ASD required repetition). Any participant who still could not respond correctly to all questions was excluded (see above).

The stimuli were images of faces displaying happy, angry and neutral expressions (Figure 1). They were generated from 20 neutral Caucasian faces (10 male) [44], with mid-range trustworthiness ratings (adults used a scale ranging from 1 “not at all trustworthy” to 9 “extremely trustworthy”; *M* = 5.0, *SD = *0.4) [45]. Each identity was morphed, using Fantamorph v5.3.1 (http://www.fantamorph.com), with three composite faces displaying happy, angry and neutral expressions respectively (each an average of 50 identities) [46]. We morphed the original (neutral expression) images towards a neutral composite, for the neutral face condition, to ensure that all stimuli were morphs. Standard morphing procedures were used to create morphs at 25% and 50% by blending the original faces with the composites in different proportions, e.g. a 25% angry morph was a 75/25 blend of an original face and the angry composite. There were 120 stimuli in total: 3 expressions (happy, angry, neutral)×2 intensities (25%, 50%)×20 identities.

On each trial, a face was presented on screen for 1500 ms for participants to rate with the number keys using a 7-point scale consisting of numbered cups (1 = not very trustworthy to 7 = very trustworthy) [47]. Faces subtended an average visual angle of 8.4°×6.3° at an approximate viewing distance of 50 cm. Each trial was initiated with a space-bar press. The 120 faces were presented in randomized order in 6 blocks of 20 trials. Between each block participants were given a break in which they were told ‘fun facts’ about Zeb the alien. Participants began with 10 practice trials: 4 trials using well-known cartoon faces (2 trustworthy, 2 male) and 6 trials using real faces (3 expressions×2 intensities). Stimuli used for practice trials were not used in the main task.

The neutral expressions were also rated on their resemblance to happy and angry facial expressions by a group of typical adults. Ratings of emotional expressions (happy, angry) were obtained using a 7-point scale (1 = not at all happy/angry to 7 = very happy/angry). Participants were informed that all faces would be emotionally neutral but could nevertheless show subtle variations in emotional information. They were encouraged to use the whole range of the scale.

#### Expression recognition

The expression recognition task was adapted from Gao and Maurer [48], [49]. Children were told that they would be shown the faces of people who were watching different movies that made them feel different things. They were told to select the expression, from five cartoon emoticon-style faces, which best matched what the person was feeling. The experimenter emphasized that there could be different intensity expressions.

The stimuli were photographs of four models (two male) from the NimStim Face Stimulus Set [50] each posing happy, angry, sad, fearful and neutral expressions. Eight levels of intensity (0, 10, 20, 30, 40, 60, 80, 100) were created for each of the four expressions by morphing between neutral (0%) expressions and 100% intensity levels in varying degrees [48]. This resulted in 128 stimuli: (4 expressions×7 intensity levels×4 models)+(4 neutral expressions×4 models).

Each trial began with a face presented on screen for 1000 ms followed by a response screen that prompted the participant to indicate the expression that corresponded to what the person was feeling. Children pointed to the appropriate icon and the experimenter pressed the corresponding key to reduce the cognitive demands of the task. There were 128 trials in total presented in randomized order. To ensure task duration was age-appropriate the task was split into two sessions. Two different models (one male) were presented in each session (64 trials in total), with order of model presentation counterbalanced between participants.

## Results

Two extreme scores defined by SPSS (one ASD 50% angry difference score and one ASD happy expression recognition threshold) were replaced with the next lowest score. Following replacement of these scores all distributions of trust and expression judgments for each group were normal and therefore appropriate for parametric analysis [51].

### Trustworthiness from Expressive Faces (Temporal Extension)

We were interested in the extent to which overt happy and angry expressions modulated trust judgments in children with ASD, relative to typically-developing children. Given the absence of any specific predictions about the relative magnitude of effects for the two expressions, we did not equate the intensity of the happy and angry expressions and we consider them separately in our analysis. In each case we used a 2×2 mixed ANOVA to investigate the effects of group (ASD, typical) and intensity (25%, 50%) on the influence of expression on trust ratings, indexed as the difference between trust ratings of expressive faces and neutral faces (see below). Descriptive statistics for each group’s trustworthiness ratings of the angry, neutral and happy expression faces are shown in Table 2.

**Table 2:** Descriptive statistics for trustworthiness ratings of the 25% and 50% angry, neutral and happy faces for each group.

	25%			50%		
	Angry	Neutral	Happy	Angry	Neutral	Happy
	*M (SD)*	*M (SD)*	*M (SD)*	*M (SD)*	*M (SD)*	*M (SD)*
ASD	3.4 (1.3)	3.7 (1.2)	4.7 (1.0)	3.1 (1.4)	3.7 (1.4)	5.4 (1.3)
Typical	3.6 (0.9)	3.9 (0.8)	4.9 (0.8)	3.1 (1.3)	4.0 (0.9)	5.5 (1.0)

#### Angry faces

**Figure 2:**
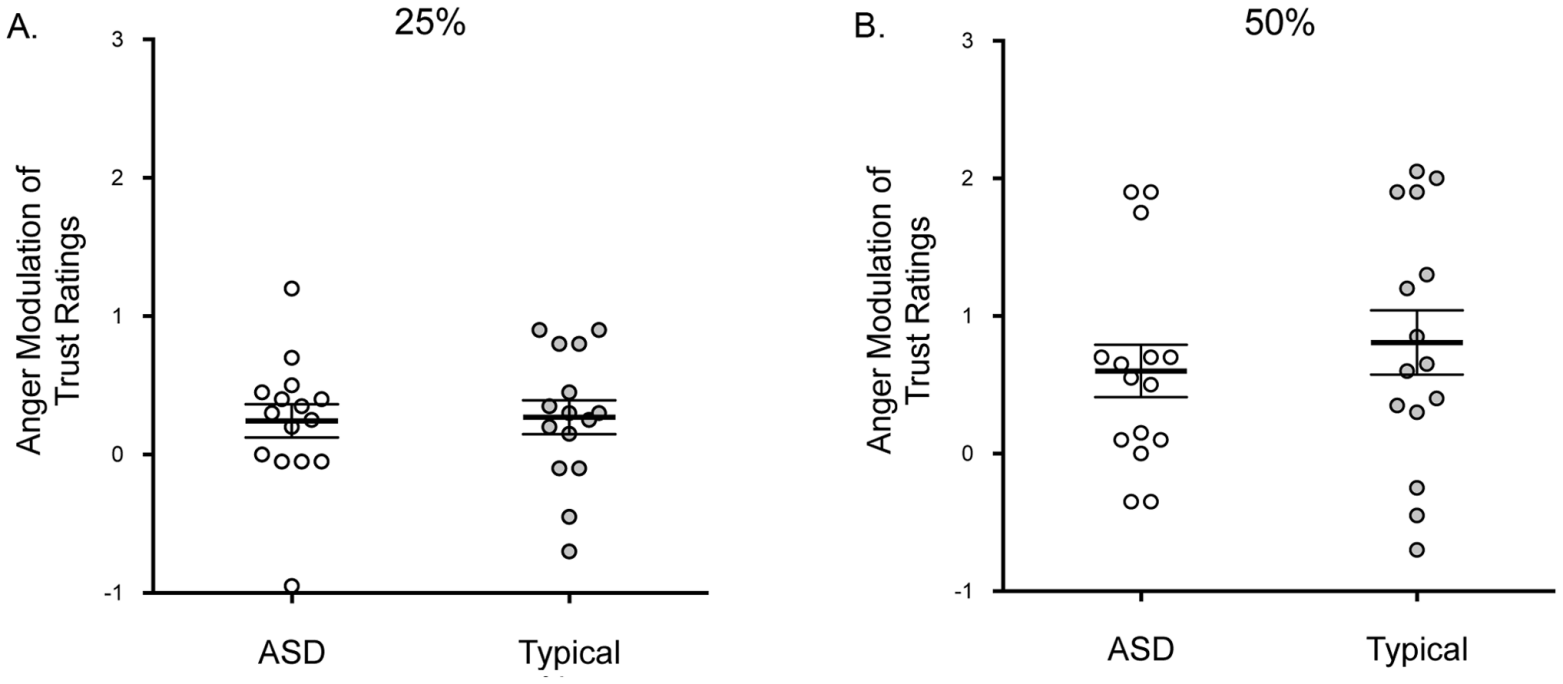
Anger modulation of trust ratings. Mean difference (SEM) in trustworthiness ratings for angry and neutral expressions at 25% (A) and 50% (B) intensity for each group. Larger values indicate greater modulation of angry expressions on trust judgments and zero indicates no modulation. Individual participants are shown.

The dependent variable was the mean difference between trustworthiness ratings of the angry and neutral faces (neutral minus angry). One sample t-tests revealed that this value was significantly greater than zero for the typically-developing children for the 25% and 50% angry faces, all *t*s >2.21, *p*s <.05, Cohen’s *d*s >1.18, which confirms that angry expressions had the predicted negative effect on perceived trustworthiness. The children with ASD also showed a significant difference from zero for the 50% angry expressions, *t*(14)* = *3.15, *p = *.007 and Cohen’s *d = *1.68, and a marginal difference from zero for the 25% expressions, *t*(14)* = *2.02, *p = *.063 and Cohen’s *d = *1.08 (Figure 2).

Our ANOVA revealed a significant main effect of intensity on the influence of anger, *F*(1, 28) = 7.58, *p = *.01, *partial* η^2^ = .21. Angry expressions influenced trustworthiness more at 50% (*M = *0.7, *SD = *0.8) than at 25% (*M = *0.3, *SD = *0.5). There was no main effect of group, *F*(1, 28) = 0.40, *p = *.53, *partial* η^2^ = .01, and no interaction of group and intensity, *F*(1, 28) = 0.31, *p = *.58, *partial* η^2^ = .01 (Figure 2).

#### Happy faces

**Figure 3:**
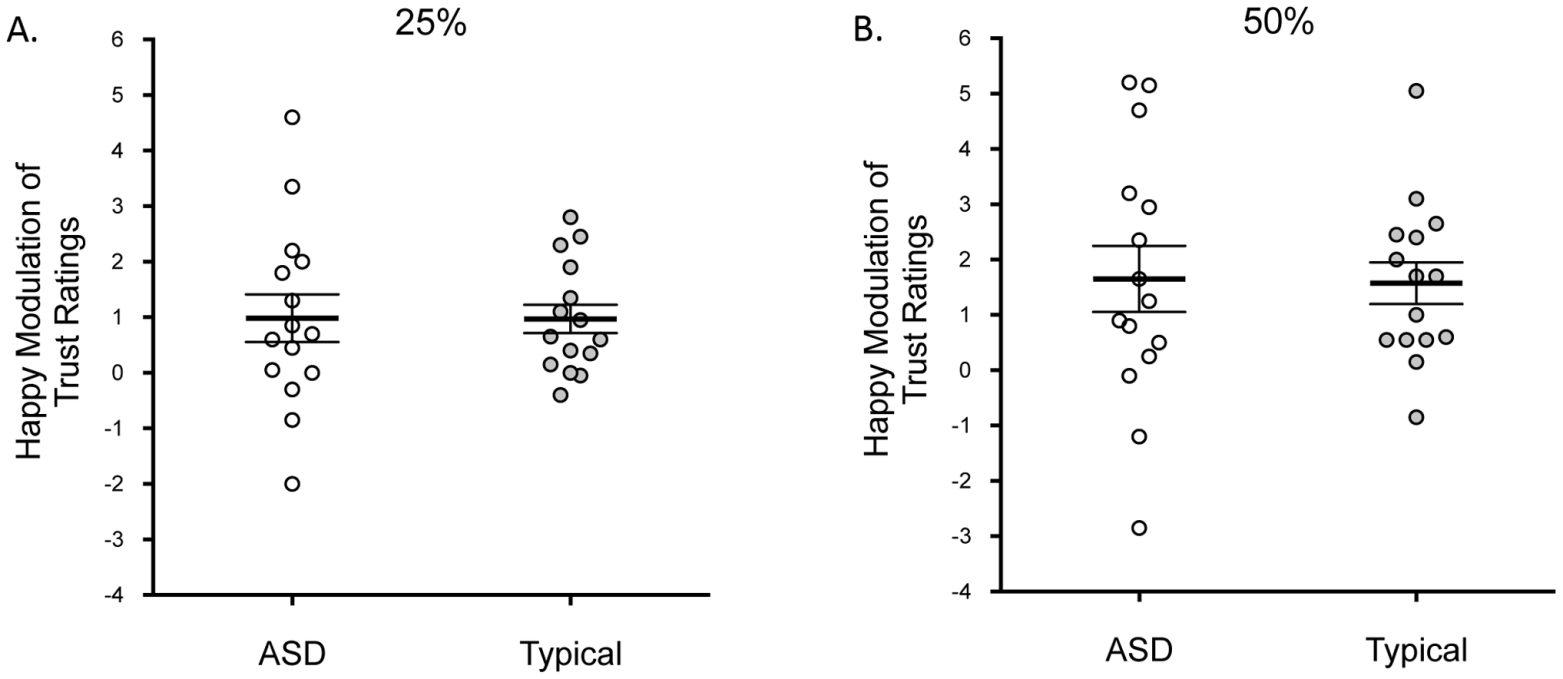
Happy modulation of trust ratings. Mean difference (SEM) in trustworthiness ratings for happy and neutral expressions at 25% (A) and 50% (B) intensity for each group. Larger values indicate greater influence of happy expressions on trust judgments and zero indicates no modulation. Individual participants are shown.

The dependent variable was the mean difference between trustworthiness ratings assigned to the happy and neutral faces (happy minus neutral). One sample t-tests indicated that this value was significantly greater than zero for children with ASD and typically-developing children at both intensities, all *t*s >2.30, *p*s <.05, Cohen’s *d*s >1.23 (Figure 3). Thus cues to happiness had a positive effect on perceived facial trustworthiness in children with and without ASD.

Our ANOVA revealed a significant main effect of intensity on the influence of happiness, *F*(1, 28) = 12.35, *p = *.002, *partial* η^2^ = .31. Happy expressions influenced trustworthiness judgments more at 50% (*M = *1.6, *SD = *1.9) than at 25% (*M = *1.0, *SD = *1.3). As with the angry cues, there was no significant difference between the groups, *F*(1, 28) = 0.006, *p = *.94, *partial* η^2^<.001, and no interaction between group and intensity, *F*(1, 28) = 0.03, *p = *.86, *partial* η^2^ = .001, indicating that the influence of happy cues on trustworthiness judgments did not differ for the children with ASD and typically-developing children (Figure 3).

We also investigated whether the influence of emotion expressions on trust judgments was associated with ASD symptomatology, as indexed by SCQ scores, in our sample of children. This analysis revealed no significant correlations between the modulatory power of 25% happy expressions, 50% happy expressions, or 50% angry expressions and SCQ scores (see Table 3). However, there was a significant positive correlation between symptom severity and the modulatory power of the 25% angry expressions. This correlation suggests that greater symptom severity was associated with greater influence of the 25% angry expressions on trust judgments.

**Table 3:** Correlations between the influence of happy and angry expressions on trustworthiness judgments and autism symptom scores in the children with ASD.

Intensity	Expression	SCQ scores (*n = *15)	
		*p*	*r*
25%	Angry	.63	.01
	Happy	−.14	.62
50%	Angry	.08	.77
	Happy	.05	.86

### Trustworthiness from Neutral Faces (Emotion Overgeneralization)

To assess whether there was also an association between perceived trustworthiness and more subtle emotional expressions for children with ASD we looked at the relationship between the children’s trustworthiness ratings and adults’ ratings of the subtle emotion cues present in the neutral expression face stimuli. The internal consistency of the adult expression ratings was high (Cronbach’s alpha = .86 and.93 for angry and happy expressions respectively) but the internal consistency of the trust ratings was low and did not support averaging across participants (Cronbach’s alpha = −.30 and −.44 for the ASD and typical group respectively). We therefore computed separate correlation coefficients for the association between trust ratings and mean expression ratings (happy, angry) of each identity for each participant. Fisher’s r to z transformation was applied to these correlation coefficients prior to analysis [52].

One-tailed, one sample t-tests indicated that the mean correlation between trust and anger ratings was significantly different from zero (i.e., negative) for children with ASD (*M* = −.10, *SD = *.20), as well as the typically-developing children (*M* = −.14, *SD = *.21), both *t*s>−1.80, *p*s <.05, Cohen’s *d*s >0.97. The mean correlation between trust and happiness ratings was significantly greater than zero for the typically-developing children (*M = *.14, *SD = *.21), *t*(14) = 2.56, *p* = .02, Cohen’s *d* = 1.37, and showed a similar trend for the children with ASD (*M = *.09, *SD = *.24), but this value did not reach significance, *t*(14) = 1.40, *p* = .09, Cohen’s *d* = 0.75. These results suggest subtle emotion cues in neutral faces influence trust ratings of children with ASD as well as typically-developing children.

A 2×2 mixed ANOVA on the trust correlations, with group (ASD, typical) as a between-participants factor and expression (angry, happy) as a within-participants factor revealed no main effect of group, *F*(1, 28) = 0.06, *p = *.81, *partial* η^2^ = .002, and no interaction between group and expression, *F*(1, 28) = 0.43, *p = *.52, *partial* η^2^ = .015. These results indicate that the children with ASD did not differ from typical children with regard to the influence of subtle emotion cues from neutral faces on trust judgments. There was a main effect of expression, *F*(1, 28) = 9.34, *p = *.005, *partial* η^2^ = .25, which reflected the difference between the negative association between trust and anger ratings (*M* = −.12, *SD = *.20) and the positive association between trust and happiness ratings (*M = *.11, *SD = *.22).

### Expression Recognition

We calculated children’s thresholds for correctly identifying happy and angry expressions on our recognition task [48], [49]. For each participant we fitted a cumulative Gaussian function to the responses for each intensity level for each expression in Graphpad Prism 5. The threshold level represents the intensity level at which the face was correctly identified as happy or angry (50% of the time). The data for two participants (one typical and one ASD) were removed due to poor fits (R^2^<.5).

**Figure 4:**
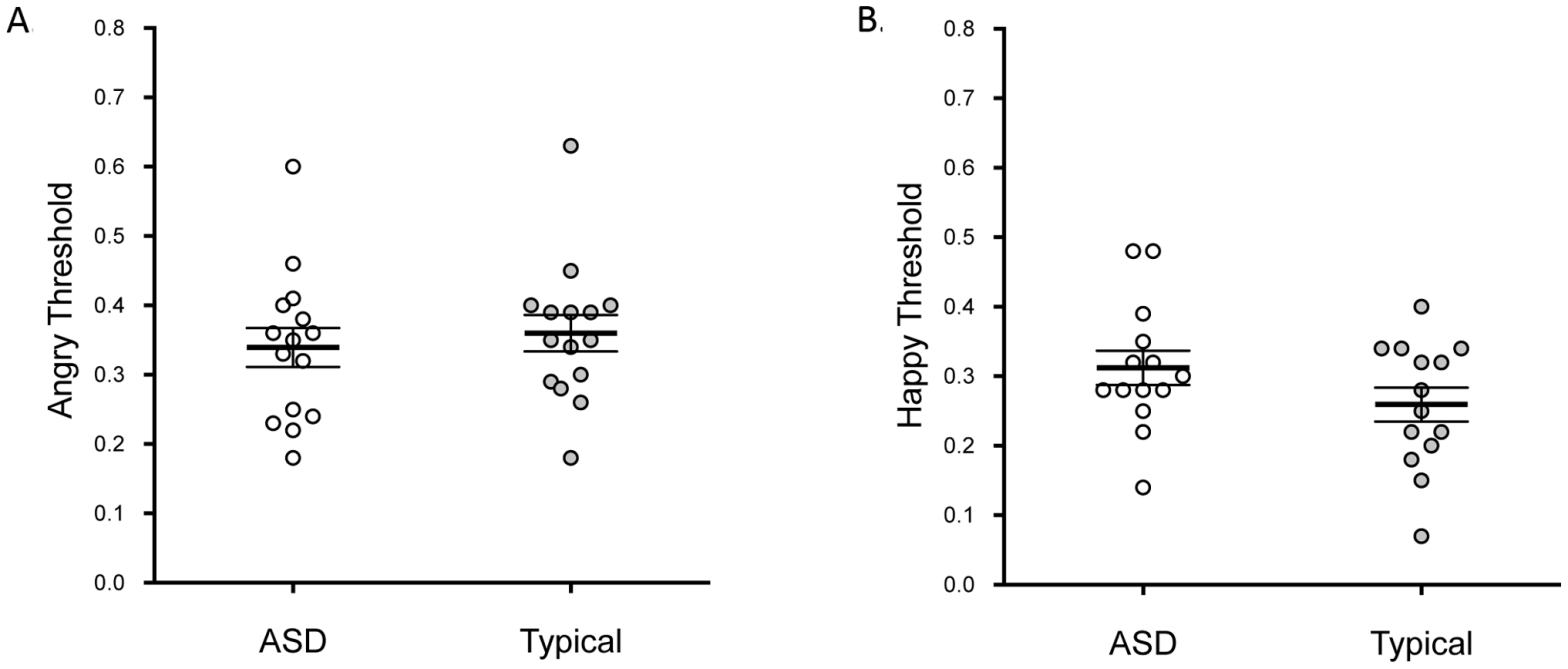
Expression recognition ability. Mean thresholds (SEM) for recognition of the angry (A) and happy (B) expressions for each group. Lower thresholds indicate greater sensitivity to the expression. Individual participants are shown.

We used a 2×2 mixed ANOVA to examine the effects of expression (happy, angry) and group (ASD, typical) on this measure of expression sensitivity. There was a significant main effect of expression, *F*(1, 26) = 5.78, *p = *.024, *partial* η^2^ = .18, with children more sensitive to the happy expressions (*M = *0.3, *SD = *0.1) than the angry expressions (*M = *0.4, *SD = *0.1). There was no main effect of group, *F*(1, 26) = 0.29, *p = *.59, *partial* η^2^ = .01, and no interaction between group and expression, *F*(1, 26) = 2.19, *p = *.15, *partial* η^2^ = .08. This indicates that the children with ASD did not differ from the typical group with respect to their sensitivity to happy and angry expressions (Figure 4).

## Discussion

We found that facial trustworthiness judgments in children with ASD were significantly modulated by happy and angry emotional expressions, like typically-developing children. These results suggest that facial expressions of emotion not only communicate emotional states but also contribute to impressions of trustworthiness for cognitively-able children with ASD.

Our findings extend previous trust perception research in ASD, which has solely focused on adults’ trust attributions [11], [17]–[21]. Here, we show that children with ASD, who may be less likely than adults to have developed compensatory mechanisms to overcome social cognitive difficulties, draw upon emotion cues when making inferences of trustworthiness from faces. Specifically, overt angry expressions diminished the appearance of trustworthiness and overt happy expressions increased the appearance of trustworthiness in faces for them. Although the effects of the 25% angry expressions did not reach significance, there was a large effect size and no group differences relative to the typical children, which suggests that the non-significant result reflected our small sample size rather than reduced modulatory power for subtle angry cues. These results suggest that trait attributions in children with ASD may reflect the temporal extension of transient facial cues as signals of more enduring characteristics.

We also provide evidence that this relationship between emotion perception and trustworthiness judgments extends to even very subtle emotion cues present in neutral faces. Our results revealed that the children with ASD did not differ from typical children with regard to the influence of subtle emotion cues from neutral faces on trust judgments. There was a negative association between trust and anger ratings and a positive association between trust and happiness ratings for both groups of children. Although the correlation between trust and happiness ratings did not reach significance for the children with ASD, there was again a moderate effect size, suggesting that this non-significant result reflects low statistical power. These results indicate that children with ASD may also be sensitive to the effects of emotion overgeneralization and provide further support for an association between emotional expressions and trust attributions in children with ASD.

Our findings are consistent with previous adult studies reporting no significant differences in trustworthiness judgments between typical adults and adults with ASD [17]–[19]. However, there are also studies that *have* reported atypicalities in response to trustworthy and untrustworthy faces in adults with ASD [11], [20], [21]. It is likely that considerable variation in participant demographics, matching strategies and task format across studies contributes to the heterogeneity of results. Moreover, differences in expression recognition ability between participant groups may also, in part, account for inconsistencies. This latter explanation seems plausible given that two of the previous studies reporting differences in trustworthiness judgments between typical adults and adults with ASD also report emotion processing impairments in the ASD group [20], [21].

Perhaps critically, the children with ASD in the current study did not exhibit expression processing impairments. Previous studies have reported expression recognition difficulties in similar samples [53], [54], [55]. However, this group of children with ASD demonstrated an intact sensitivity to happy and angry expressions on our measure of expression recognition, which was designed to be sensitive to detect subtle processing atypicalities. Our study is not the first to find intact expression processing in individuals with ASD [56], [57]–[60]. Indeed, despite considerable research attention the literature remains divided as to whether or not individuals with ASD show reliable emotion processing impairments [32]–[35]. Based on our findings, we can conclude that when expression processing is intact in children with ASD, emotional expressions influence impressions of trustworthiness from faces.

Clearly, however, an important extension of the current study will be to investigate trust perception in children with ASD who *do* show significant emotion processing impairments. Such research would allow us to determine whether the modulatory power of emotion cues on trust judgments can be dissociated from expression recognition ability. It seems likely that impairments reading expression information would limit the influence of these cues on trustworthiness judgments. However, it is also possible that the modulation of trait judgments by emotion cues may be independent of explicit expression recognition ability, particularly given the automatic nature of these appearance-based trait inferences [4], [61].

Interpretation of the current results must take into consideration the characteristics of our cognitively-able ASD sample. Though low levels of *current* autism symptoms in a subset of our sample, as indexed by scores on the ADOS-2 [40], could limit the generalizability of our findings, we highlight that all participants had received an ASD diagnosis from a multidisciplinary team and were scored above the criterion for ASD on a well-validated retrospective symptom measure (SCQ) [38]. In addition, our sample demonstrated other perceptual atypicalities in face perception, e.g. significant impairments in face recognition, relative to typical children, as indexed by scores on the CFMT-C. Moreover, when we reanalyzed our data including only those children with ASD who met the more conservative current symptom criterion (score of 7 or above on the ADOS-2), the modulatory effects of the happy and angry expressions remained in the predicted direction, with moderate-large effect sizes across all conditions.

Interestingly, results of our correlational analysis between symptom severity and the influence of emotion expressions on trust judgments suggested that children with more severe ASD symptomatology may *overuse* certain expression cues. Our results revealed a positive correlation between symptom severity and the modulatory power of 25% angry expressions on trust judgments. That is, more severe ASD symptoms were associated with greater influence of the 25% angry expressions when judging trustworthiness. This overuse of the angry expression cues may constitute evidence of atypical trust perception. It would be interesting to see whether the same profile of trust perception is observed in a larger sample of children with more severe ASD symptomatology.

The current study adds to a growing body of evidence detailing intact aspects of social cognition in individuals with ASD. Other studies have revealed that adults with ASD spontaneously infer traits, such as whether a person is clever, honest and friendly, from descriptions of behaviour [62] and children with ASD have been shown to use social stereotypes to predict behaviour [63], [64]. Together these findings suggest that, despite significant impairments in interpersonal understanding, social knowledge is not universally disrupted in individuals with ASD. Our findings suggest that trust perception may be another spared social capacity in ASD. Continued research into other social judgments and trait attributions will help further our understanding of the complex profile of social cognitive ability and impairment in individuals with ASD.

In conclusion, we have shown that impressions of trustworthiness from facial appearances in cognitively-able children with ASD are significantly modulated by emotional expressions. Angry expressions diminished the appearance of trustworthiness and happy expressions increased the appearance of trustworthiness for the children with ASD, just like for the typically-developing children. The associations between perceived emotion and trustworthiness also extended to subtle expression cues present in neutral faces. These findings indicate that cognitively-able children with ASD draw upon emotion cues when judging facial trustworthiness, which suggests that similar mechanisms may drive trustworthiness inferences in typical children and children with ASD.

## Supporting Information

Appendix S1 **This supporting information describes the results for each task when the data were reanalysed with only those children with ASD who scored above the ADOS-2 cut-off for** ***current*** **ASD symptoms (** ***n =*** **9).**
(DOCX)
