# Supplementary material for: Facial Trustworthiness Judgments in Children with ASD Are Modulated by Happy and Angry Emotional Cues
Source: PLoS One. 2014 May 30;9(5):e97644. doi: 10.1371/journal.pone.0097644 (PMC4039438; doi:10.1371/journal.pone.0097644)
Supplement: Appendix S1 — This supporting information describes the results for each task when the data were reanalysed with only those children with ASD who scored above the ADOS-2 cut-off for current ASD symptoms ( n = 9). (DOCX) [file pone.0097644.s001.docx]

**Supporting Information S1**

This supporting information describes the results for each task when the data were reanalysed with only those children with ASD who scored above the ADOS-2 cut-off for *current* ASD symptoms (*n* = 9). The data from this restricted sample of children with ASD was compared against the previously matched sample of typically-developing children (*n* = 15). These two groups did not differ significantly in terms of chronological age, non-verbal IQ or full-scale IQ, all *t*s < 1.06, *p* > .31, Cohen’s *d*s < 0.62.

**Trustworthiness from Expressive Faces (Temporal Extension)**

One sample t-tests indicated that the modulatory effects of angry and happy expressions on trustworthiness judgments were no longer significantly greater than zero for the children with ASD at 25% (Angry: *M* = 0.2, *SD* = 0.6; Happy: *M* = 0.8, *SD* = 1.7) or 50% (Angry: *M* = 0.4, *SD* = 0.7; Happy: *M* = 1.4, *SD* = 2.7), all *t*s < 1.59, *p*s > .15, Cohen’s *d*s > 0.86. However, there were still large effects sizes for all conditions suggesting that the non-significant results reflect diminished statistical power rather than a meaningful difference in this more conservatively characterised sample of children with ASD.

As with the main analysis, a 2 x 2 mixed ANOVA revealed no main effect of group (ASD: *M* = 0.3, *SD* = 0.5; Typical: *M* = 0.5, *SD* = 0.5), *F*(1, 22) = 1.28, *p* = .27, *partial* η^2^ = .06, or interaction between group and intensity, *F*(1, 22) = 1.18, *p* = .29 *partial* η^2^ = .05, for the angry faces, as well as no main effect of group (ASD: *M* = 1.1, *SD* = 2.2; Typical: *M* = 1.3, *SD* = 1.2), *F*(1, 22) = 0.06, *p* = .82, *partial* η^2^ = .002, or interaction between group and intensity, *F*(1, 22) = 0.015, *p* = .90, *partial* η^2^ = .001, for the happy faces.

**Trustworthiness from Neutral Faces (Emotion Overgeneralization)**

One-tailed, one-sample t-tests indicated that the mean correlations between trust and expression ratings for children with ASD were no longer significantly different from zero for anger (*M* = -.09, *SD* = .25), *t*(8) = -0.98, *p* = .18, Cohen’s *d* = .70, or happiness (*M* = .06, *SD* = .30), *t*(8) = 0.54, *p* = .30, Cohen’s *d* = .38, following the exclusion of children scoring below the ADOS-2 cut-off for current ASD symptoms. However again, there were moderate effect sizes and the trends remained in the predicted direction: a negative association between trust and anger ratings and a positive association between trust and happiness ratings. A 2 x 2 mixed ANOVA confirmed no main effect of group (ASD: *M* = -.01, *SD* = .07; Typical: *M* = .001, *SD* = .07), *F*(1, 22) = 0.27, *p* = .61, *partial* η^2^ = .01, and no interaction between group and expression, *F*(1, 22) = 0.53, *p* = .47, *partial* η^2^ = .02.

**Expression Recognition.**

When the expression recognition data were reanalysed using the more conservatively characterised sample of children with ASD, the absence of a group effect persisted (ASD: *M* = 0.3, *SD* = 0.07; Typical: *M* = 0.3, *SD* = 0.07), *F*(1, 21) = 0.002, *p* = .97, *partial* η^2^ < .001, as did the absence of an interaction between group and expression, *F*(1, 21) = 1.55, *p* = .23, *partial* η^2^ = .07.
